# Predictive value of preoperative Fried Frailty Phenotype assessment and serum biomarkers on the prognosis of elderly patients with femoral neck fracture under general anesthesia within 3 months after surgery

**DOI:** 10.55730/1300-0144.5883

**Published:** 2023-12-11

**Authors:** Fu XU, Xin KUANG, Baofeng CAO, Yang YUE

**Affiliations:** 1 Department of Anesthesiology, Shenzhen Longhua District People’s Hospital, Shenzhen City, China; 2 Department of Obstetrics, Longhua District Maternity and Child Health Hospital, Shenzhen City, China

**Keywords:** Fried Frailty Phenotype, femoral neck fracture, FGFR3, RUNX2, prognostic value

## Abstract

**Background/aim:**

Femoral neck fracture (FNF) seriously impact the health of the elderly and affect their long-term quality of life of the patients. This study aimed to determine whether combining the preoperative Fried Frailty Phenotype (FFP) with serum fibroblast growth factor receptor 3 (FGFR3) and run-related transcription factor 2 (RUNX2) could better predict the prognosis of elderly patients with FNF 3 months after surgery.

**Materials and methods:**

A total of 150 elderly patients with FNF (60–89 years old) were enrolled and divided into a nonfrailty cohort and a frailty cohort based on preoperative FFP evaluation. The hip recovery of patients 3 months after surgery was evaluated using Harris Hip Score (HHS). Serum FGFR3 and RUNX2 levels were analyzed, and the relationship between HHS and serum FGFR3 and RUNX2 levels was evaluated. The specificity and sensitivity of FFP, serum FGFR3 and RUNX2 were evaluated using ROC curves before surgery. Potential prognostic factors were analyzed using multivariate logistic regression.

**Results:**

Serum FGFR3 and RUNX2 levels were lower and hip recovery was poorer in the frailty cohort than in the nonfrailty cohort (p < 0.001). Within 3 months after surgery, there were 12 deaths (17.6%) in the frailty cohort and 1 in the nonfrailty cohort (1.2%) (p < 0.001). FFP assessment combined with serum FGFR3 and RUNX2 levels had a higher diagnostic significance. Readmission and preoperative frailty phenotype were independent factors affecting the prognosis of patients with FNF. HHS scores greater than 70 and higher levels of serum FGFR3 and RUNX2 cutoff values (7.85 ng/mL and 56.5 ng/mL, respectively) were identified as protective factors for prognosis.

**Conclusion:**

Assessing FFP alongside serum FGFR3 and RUNX2 levels may aid in evaluating the prognosis of elderly patients with FNF 3 months after surgery.

## 1. Introduction

Femoral neck fracture (FNF) poses a serious threat to the health of the elderly due to its high morbidity and mortality and causes a huge economic burden [1]. Elderly hip fractures are mainly related to osteoporosis, among which FNF is the most common [2]. As the global population continues to age, more than 1 million hip fractures are reported each year worldwide, with this number particularly high in developing countries. It has been reported that by 2050, there will be more than 4 million hip fractures worldwide, of which 1.5 million will occur in China [3], while another review reported that the mortality rate within 1 year of hip fractures in older adults may be between 14% and 58% [4]. Currently, common clinical methods for FNF in the elderly are internal fixation, artificial femoral head replacement, hip replacement, etc. [5, 6]. General anesthesia is usually administered to patients with FNF, but during the induction of general anesthesia, a variety of drugs are required, which can affect the respiratory and circulatory functions of patients, cause significant fluctuations in heart rate and blood pressure, increase cardiac load and work, and easily lead to cardiovascular adverse events such as tachycardia and hypotension [7, 8]. In addition, elderly patients are particularly susceptible to hemodynamic fluctuations during surgery and anesthesia stimulation, which can further affect patient prognosis [9]. Therefore, preoperative prognostic evaluation is often carried out in elderly patients to better guide clinical treatment, predict postoperative complications, and improve prognosis. Frailty is a state in which the human body is vulnerable to damage after experiencing stressful events due to the decline in the functional reserves of multiple systems [10]. Frailty assessment was first used to evaluate the physiological state and survival status of elderly people in communities, and frailty is an independent predictor of postoperative complications, prolonged hospital stay, death, and other adverse prognosis [11]. Fried Frailty Phenotype (FFP) is a classic method for frailty assessment. It is simple to operate and widely used in clinical and research studies. The scale takes frailty as a precursor state of clinical events and can independently predict adverse events so that preventive measures can be taken [12]. However, no studies have reported the predictive value of FFP in the 3-month prognosis of elderly patients with FNF under general anesthesia.

Fibroblast growth factor receptor 3 (FGFR3) is one of the four typical high-affinity receptors for FGF ligands [13]. FGFR3 in periosteal cells drives the transformation of cartilage into bone in bone repair [14]. Recombinant FGFR3 therapy restores the effective maturation of growth plate chondrocytes in bone and promotes bone growth in a dose-dependent manner [15].

Run-related transcription factor 2 (RUNX2) is considered significant in the maturation of chondrocytes and can promote the transcription of various mineralization-related protein genes in osteocytes [16]. The homeostasis of bone tissue requires strict regulation of multiple signaling pathways, and RUNX2-dependent bone development or bone formation involves a complex regulatory cascade. It is reported that RUNX2 can improve the maintenance of the osteoblastic phenotype of mesenchymal stem cells and promote bone repair of femoral head necrosis [17].

The elderly have poor tolerance to anesthesia and surgery, and preoperative evaluation can effectively predict the prognosis and provide a basis for clinical treatment. Although poor prognosis in patients with FNF has been well documented, prognostic factors have not been thoroughly examined. Identifying which factors are associated with prognosis may help surgeons make treatment decisions and ultimately enhance care for patients with FNF. The objective of this study was to determine whether combining preoperative FFP with serum FGFR3 and RUNX2 could better predict the prognosis of elderly patients with FNF 3 months after surgery.

## 2. Materials and methods

### 2.1. Research subjects

All participants, including FNF patients and healthy controls, provided informed consent. The inclusion criteria were as follows: 1) FNF diagnosed by clinical and hip X-ray examination; 2) age ≥60 years; 3) ability walk normally before the fracture; 4) absence of cognitive dysfunction; 5) surgery performed under general anesthesia.

The exclusion criteria were as follows: 1) presence of malignant tumors; 2) pathological hip fracture; 3) unwillingness to receive surgical treatment; 4) old hip fracture; 5) ipsilateral hip fracture history or surgical history; 6) Incomplete clinical data.

This was a central study in Shenzhen Longhua District People’s Hospital, and data were collected prospectively. From December 2018 to December 2022, 150 patients with FNF were enrolled, with a mean age of 74 years (95% confidence interval [CI]: 63–85).

Before anesthesia and surgery, the ASA score was used to determine each patient’s physical state. The ASA score, assessed by the senior anesthesiologist responsible for the surgery, categorizes patients as: I (healthy patients), II (patients with mild systemic disease), III (patients with serious systemic disease who are not incapacitated), and IV (patients with disabling systemic diseases).

The duration of the procedure (in minutes), the estimated amount of surgical blood loss (in milliliters), and the length of the patient’s hospital stay (in days) were recorded. The patients were followed up for 3 months after the surgery, and the incidence of all-cause readmission, total postoperative complications (postoperative infection, cardiovascular and cerebrovascular accidents, abnormal liver function, postoperative delirium, postoperative bleeding, lower extremity venous thrombosis, electrolyte disturbance, hypoproteinemia, etc.) and total mortality were recorded.

Harris Hip Score (HHS) was used to evaluate the hip function of the patients 3 months after surgery. This assessment includes four items: pain, function, joint motion, and deformity. Two professional orthopedic surgeons scored these items, and the average score was considered the patient’s hip performance score. A score of less than 70 indicated poor recovery. Additionally, 38 age- and sex-matched subjects were recruited as the control group.

### 2.2. Preoperative frailty assessment

Preoperative FFP assessment includes the following criteria: 1) Slow step: Patients are instructed to walk 5 m at a normal speed; 2) Decreased grip strength: the maximum grip strength of the patient’s dominant hand was measured; 3) Low physical activity: Based on the International Physical Activity Questionnaire, weekly metabolic equivalents below 600 was considered low activity; 4) Fatigue: Patients were asked about two items from the depression scale, “I feel it is difficult to do anything”, “I can’t get up to do things”; 5) Low weight: unintentional weight loss of ≥5% in the past 1 year. Frailty is defined as meeting three of the above criteria. This study stipulates that meeting any criterion of the above scale is recorded as level 1, meeting two criteria as level 2, and the highest is level 5.

### 2.3. Blood sample collection

In control subjects, blood samples were collected during blood drawing for other specified medical reasons, such as anemia assessment or prior to elective surgery. Blood samples were taken from all subjects after fasting overnight and processed within 2 h. To obtain the serum, the blood was placed in an EDTA-free tube, and 10 mL of sample was centrifuged at 1300 × *g* at 4 °C for 20 min. The serum was then equally divided into 0.5-mL tubes and stored at −80 °C until analysis was performed.

### 2.4. Clinical feature collection and laboratory testing

Clinical features and anthropometry were recorded during clinical visits or through review. Clinical information included sex, age, height, weight, and disease history.

Samples were tested by the Longhua District Maternity and Child Health Hospital’s central laboratory. Serum FGFR3 and RUNX2 were determined using ELISA kits, purchased from RD Systems Inc. All samples were repeated in one assay to avoid interassay variation. ELISA measured less than 3% intraassay variation.

### 2.5. Data statistics

Data for subjects’ clinical and anthropometric continuous variables were expressed as median (25th and 75th percentiles), while categorical variables were expressed as frequency (%). Enumeration data were measured by chi-square or Fisher’s exact test between groups. Measurement data were compared between two groups with the Mann–Whitney U test, and between multiple groups with the Kruskal–Wallis H test. The correlation between HHS and serum FGFR3 and RUNX2 levels was assessed using Spearman’s correlation coefficient. The predictive value of preoperative FFP assessment on patients’ death, readmitted status, and hip recovery 3 months after surgery was determined based on the ROC curve. The area under the curve (AUC) was calculated, and the cutoff value was determined using the Youden index. Univariate binary logistic regression was used to screen the prognostic factors. Multivariate logistic regression was used to analyze prognostic factors, including variables that showed statistical effects in univariate variables, to study the prognostic value of preoperative FFP assessment, and serum FGFR3 and RUNX2 levels. A p-value below 0.05 was considered statistically significant. SPSS software 22.0 was employed for analysis, and GraphPad Prism 8.3.0 (GraphPad, San Diego, CA) for mapping.

## 3. Results

### 3.1. Baseline data and operative status of elderly patients with FNF

Table 1 summarizes the demographic data of a total of 150 elderly patients with FNF, most of whom were 110 women (73.3%) and 40 men (26.7%), with a median age of 67 years and a median body mass index (BMI) of 21.8 kg/m^2^. Most patients had a Grade II or III ASA classification at baseline (78.0%). According to the preoperative FFP assessment method, we divided the patients into two categories: 82 cases (54.7%) of nonfrailty and 68 cases (45.3%) of frailty.

We observed a significant difference in ASA classification between the two cohorts (p = 0.005), with 23 (33.8%) and 11 (16.2%) patients in grade III and IV, respectively. Conversely, patient age, sex, BMI, and disease history did not differ between the two groups. As shown in Table 2, we did not find a difference in the duration of surgery and the amount of intraoperative blood loss between the nonfrailty and frailty groups, and frailty patients had a longer hospital stay than the nonfrailty group (p = 0.013), with a median of 17 days. In the total patient cohort, the 3-month readmission rate and complication rate were 24% and 28%, respectively, and the frailty cohort had a higher readmission rate and complication rate than the non-frailty cohort (p < 0.001, p = 0.005). Three months after surgery, the hip recovery of patients was assessed using HHS, and it was found that the majority of patients who were evaluated as frailty phenotype before surgery had poor recovery (58.8%) (p < 0.001). Notably, there were 12 deaths in the frailty cohort (17.6%) and only 1 in the nonfrailty cohort (1.2%) within 3 months after surgery (p < 0.001). These results suggest that preoperative FFP assessment to determine whether patients have frailty phenotype may have a certain guiding effect on postoperative readmission, complication rate, hip recovery, and 3-month risk of death.

### 3.2. Preoperative FFP assessment combined with serum FGFR3 and RUNX2 levels can effectively predict the prognosis of elderly patients with FNF 3 months after surgery

**Figure 1:**
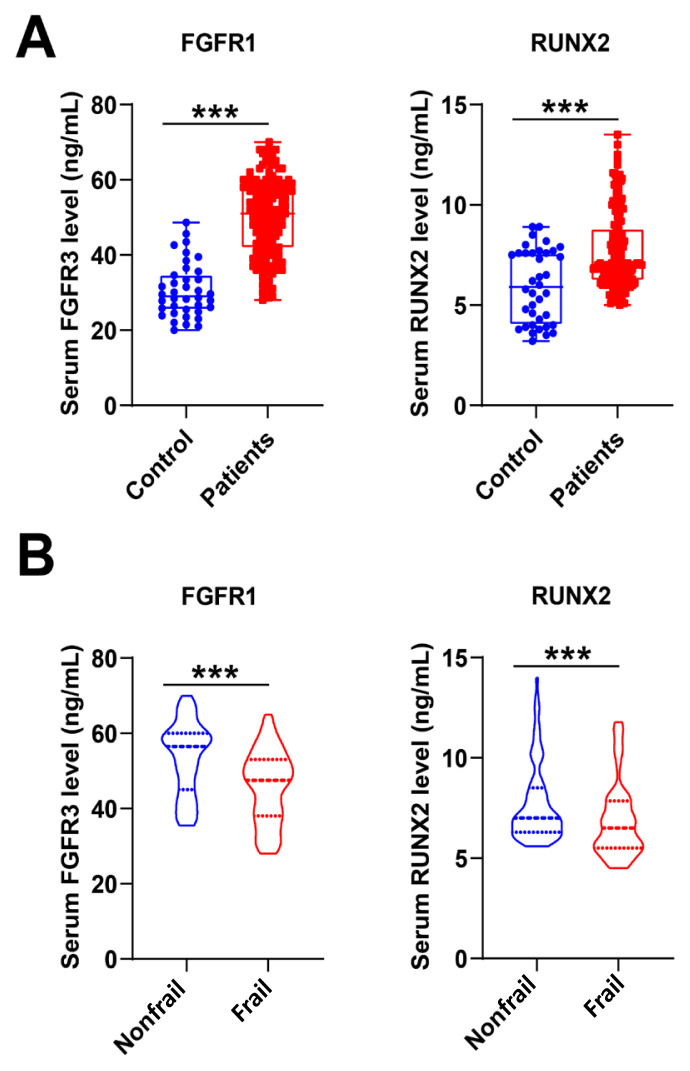
(A) Comparison of healthy controls with elderly patients with FNF. (B) Comparison of nonfrailty and frailty elderly patients with FNF. *** p < 0.001; ** p < 0.01; * p < 0.05.

**Figure 2:**
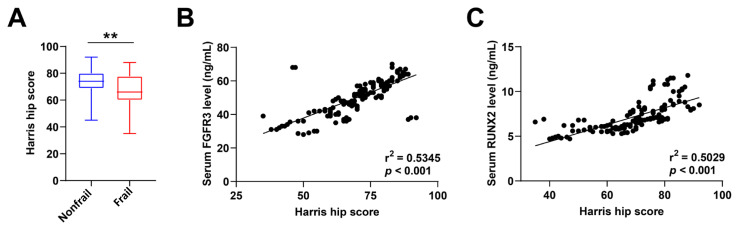
(A) HHS in patients with and without frailty. The Spearman test was used to determine the correlation between HHS and serum (B) FGFR3 and (C) RUNX2 levels. Preoperative frailty and nonfrailty HHS were evaluated using the Mann–Whitney U test. p < 0.05 is considered significant.

**Figure 3:**
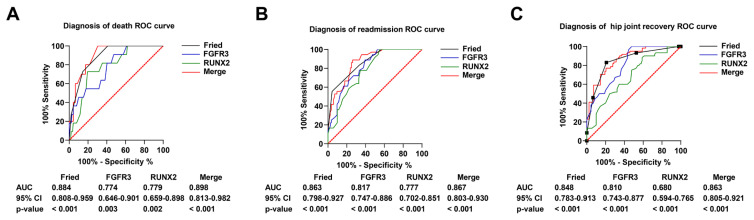
ROC curve of preoperative FFP combined with serum FGFR3 and RUNX2 levels to predict (A) death, (B) readmission, and (C) hip function in elderly patients with FNF within 3 months after surgery. p < 0.05 is considered significant.

First, we compared serum FGFR3 and RUNX2 levels and found that serum FGFR3 and RUNX2 levels were higher in patients with FNF than in controls (Figure 1A, p < 0.001). There were significant differences in FGFR3 and RUNX2 levels between control, nonfrailty, and frailty groups (Figure 1B; Table 3, p < 0.001, p = 0.002). Next, we analyzed HHS in the frailty versus nonfrailty patient cohorts, with higher HHS 3 months after surgery in the nonfrailty patients (Figure 2A, p < 0.001). In addition, Spearman’s correlation analysis showed that HHS was positively correlated with serum FGFR3 and RUNX2 levels (Figures 2B and 2C, r^2^ = 0.5345, p < 0.001; r^2^ = 0.5029, p < 0.001). The ROC curve and the AUC (Figure 3) showed that preoperative FFP assessment, serum FGFR3, and RUNX2 levels had high diagnostic values for death (Figure 3A), readmission (Figure 3B), and hip recovery (Figure 3C) of patients. Among them, the AUC value of FFP combined with serum FGFR3 and RUNX2 levels was higher than that of FFP assessment or serum FGFR3 and RUNX2 levels. It suggested that FFP assessment combined with serum FGFR3 and RUNX2 levels had better diagnostic significance.

**Figure 4:**
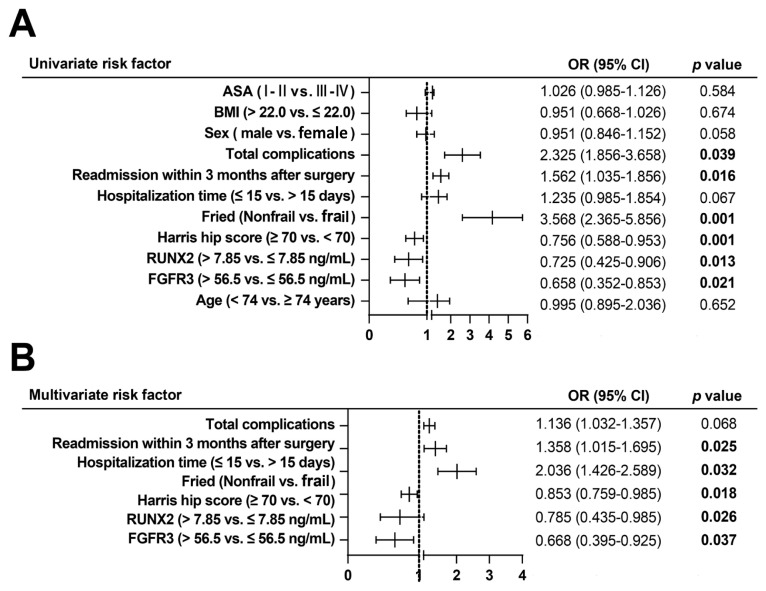
Variate analysis of univariate and multivariate analyses affecting hip functional recovery 3 months after surgery in elderly patients with FNF. p < 0.05 is considered significant.

The cutoff value of serum FGFR3 and RUNX2 obtained by Youden were calculated based on the patient ROC curve for stratification. To determine prognostic factors in elderly patients with FNF, multivariate binary logistic regression analysis was performed (Figure 4), and variables showing significance in univariate analysis were used as covariates. Patient baseline characteristics, including age, sex, and BMI, were used as adjusting factors for regression analysis (Figure 4A). Readmission within 3 months after surgery and preoperative assessment of FFP were independent factors affecting the prognosis of patients. We did not observe postoperative complications as an independent prognostic factor. HHS (>70 scores) and higher levels of serum FGFR3 and RUNX2 cutoff values (7.85 ng/mL and 56.5 ng/mL) were protective factors for prognosis (Figure 4B).

## 4. Discussion

We investigated potential prognostic factors 3 months after surgery in elderly patients with FNF. The vast majority of patients who died 3 months after surgery had a frailty phenotype. The frailty phenotype assessed before surgery and the readmission rate within 3 months after surgery were identified as independent factors affecting the prognosis of patients with FNF. Additionally, HHS scores exceeding 70, along with higher levels of serum FGFR3 and RUNX2 cutoff values (7.85 ng/mL and 56.5 ng/mL) were observed as protective factors for prognosis. Conversely, the incidence of postoperative complications was not found to be an independent factor affecting prognosis.

In this analysis, elderly patients with FNF were selected as the investigation subjects. Due to the global aging trend and the fact that FNF is a common fracture in the elderly population, it is of high socioeconomic importance [18]. More studies have investigated the association between FNF and other diseases and the risk of death in older adults. For example, in patients with end-stage renal disease requiring dialysis, comorbidities and postoperative complications are factors contributing to the risk of readmission and death [19]. Patients with FNF combined with cancer or cardiovascular disease have an increased risk of death within 3 years [20]. In our study, however, we did not find differences in comorbidities (including hypertension, diabetes, cardiovascular and cerebrovascular disease, lung disease, and kidney disease) between the frailty and nonfrailty FNF cohorts. The ASA score, used to assess the overall physical fitness or disease of patients before surgery, is regarded as a scale to predict risk [21]. This score is associated with longer hospital stays and 30-day mortality in elderly patients with FNF [22]. Our results also showed that the frailty in preoperative FFP assessment was associated with a high ASA score. However, we did not observe an association between ASA scores and outcomes at 3 months after surgery in patients with FNF. In a study, Hirohisa et al. employed the FFP as a means to assess the relationship between frailty and postoperative complications in individuals with curative colorectal inflammation. The findings revealed a significant association between patients diagnosed with frailty and advanced age, severe postoperative complications, as well as an extended duration of hospitalization [23]. Similarly, our research results also show that postoperative hospital stay, readmissions rate, total complication rate, and mortality were correlated with preoperative FFP. In addition, FFP can also distinguish well between death, readmission, and hip recovery in patients with FNF 3 months after surgery.

FGFR3 and RUNX2 have been investigated to promote the bone repair process [13, 24, 25]. Our study was the first to combine FFP with serum FGFR3 and RUNX2 to evaluate the prognosis of patients with FNF 3 months after surgery. The organs or tissues of the human body, including bones, initiate certain self-repair after injury, which is a natural process common to all living organisms [26]. As expected, serum FGFR3 and RUNX2 levels were higher in patients with FNF, and bone repair processes were present in vivo. Serum FGFR3 and RUNX2 levels were lower in the frailty cohort than in the nonfrailty cohort. Although no significant difference in data was observed, the frailty cohort may have been affected by age, body mass index, or other unknown diseases on a physiological basis that reduces the initiation of repair processes. HHS is a disease-specific health status scale often used to measure the outcome of total hip replacement [27]. Furthermore, the research conducted by Jasvinder et al. indicates that a diminished postoperative HHS score can serve as a predictive factor for the likelihood of revision following total hip replacement, thus signifying an unfavorable prognosis for patients [28]. Our study found that HHS was higher in the nonfrailty cohort and that HHS in patients with FNF had a significant positive association with serum FGFR3 and RUNX2 levels. Furthermore, serum FGFR3 and RUNX2 levels were good differentiators of death, readmission, and hip recovery 3 months after surgery in patients with FNF. Moreover, FFP combined with serum FGFR3 and RUNX2 levels had higher diagnostic significance. There is a prevailing belief that early intervention in the advancement of weakness yields greater success in impeding or reversing its progression, thereby significantly impacting the prognosis of the disease. In our study, multivariate logistic regression confirmed that readmission within 3 months after surgery and frailty phenotype were independent factors affecting the prognosis of patients with poor hip joints. HHS scores exceeding 70 and higher levels of serum FGFR3 and RUNX2 cutoff values (7.85 ng/mL and 56.5 ng/mL) were found to be protective factors for prognosis. In addition, postoperative complications were not observed as an independent factor affecting prognosis.

## 5. Limitation

A limitation of our study is the relatively small cohort size, which may have affected the validity of the statistical analysis. Additionally, it is necessary to confirm the relationship between FFP assessment and serum FGFR3 and RUNX2 in other cohorts. In addition, more reasonable grouping should be further combined with other frailty assessment methods, such as Frailty Index. Another limitation is that the follow-up time is limited and longer studies are needed to confirm our findings.

## 6. Conclusion

Preoperative FFP assessment has a good predictive ability for postoperative adverse outcomes. FFP assessment and serum FGFR3 and RUNX2 levels were associated with prognosis in elderly patients with FNF. FFP combined with serum FGFR3 and RUNX2 to predict the prognosis of elderly patients with FNF could help clinicians identify patients with poor prognosis at an early stage and recommend better preoperative or postoperative care to minimize mortality and readmission.

## Figures and Tables

**Table 1:** Demographic data of elderly patients with femoral neck fracture.

Parameter (n%)	Nonfrail (n = 82)	Frail (n = 68)	p-value
**Age (years)**	68 (62–86)	66 (62–84)	0.252
**Sex (male/female)**	20/62	20/48	0.489
**BMI (kg/m** **^2^** **)**	22.3 (21.9–28.6)	21.5 (20.5–27.9)	0.686
**ASA classification**			
I	8 (9.7)	2 (2.9)	0.012*
II	42 (51.2)	32 (47.1)	
III	30 (36.6)	23 (33.8)	
IV	2 (2.4)	11 (16.2)	
**Medical history**			
Hypertension	38 (46.3)	27 (39.7)	0.414
Diabetes mellitus	33 (40.2)	29 (42.6)	0.766
Coronary artery	13 (30.9)	19 (27.9)	0.072
Cerebrovascular	16 (19.5)	16 (23.5)	0.55
Lung disease	8 (9.7)	9 (13.2)	0.503
Kidney disease	3 (3.6)	6 (8.8)	0.185

Data are expressed as the median (25th, 75th percentile) or number of cases (%). Enumeration data were evaluated with the chi-square or Fisher’s exact test, while measurement data were assessed using the Mann–Whitney U test to evaluate the demographics of elderly patients undergoing surgery for femoral neck fracture.

* p < 0.05 is considered significant.

**Table 2:** Comparison of surgical and postoperative outcomes of patients with frailty phenotype and nonfrailty phenotype based on preoperative assessment of Fried Frailty Phenotype scale.

Index (n%)	Nonfrail (n = 82)	Frail (n = 68)	p-value
**Operation time (min)**	79 (45–105)	82 (43–108)	0.728
**Estimated Blood lose (mL)**	52 (32–86)	50 (29–75)	0.056
**Hospitalization time (days)**	13 (6–20)	17 ((10–28)	0.013*
**Readmission within 3 months**	10 (12.2)	26 (38.2)	<0.001*
**Total complications**	17 (20.7)	25 (36.8)	0.029*
**Harris Hip Score**			
recovered well (70–100)	62 (75.6)	28 (41.2)	<0.001*
poor recovery (<70)	20 (12.2)	40 (58.8)	
**Death within 3 months after surgery**	1 (1.2)	12 (17.6)	<0.001*

Data are expressed as the median (25th, 75th percentile) or number of cases (%). Enumeration data were evaluated with the chi-square or Fisher’s exact test, while the measurement data were evaluated using the Mann–Whitney U test.

* p < 0.05 is considered significant.

**Table 3:** Serum FGFR3 and RUNX2 levels.

Index	Control (n = 38)	Nonfrail (n = 82)	Frail (n = 68)	p-value
**FGFR3 (ng/mL)**	29.1 (25.6–34.3)	56.6 (45.3–60.0)	47.5 (38.0–53.0)	<0.001*
**RUNX2 (ng/mL)**	5.9 (4.1–7.6)	7.0 (6.3–8.5)	6.5 (5.5–7.8)	0.002*

The Kruskal–Wallis H test was performed for data comparisons.

* p < 0.05 is considered significant.

## Data Availability

The datasets used and/or analyzed during the present study are available from the corresponding author on reasonable request.
